# Artificial Intelligence in Medicine: Moving From “Prediction” to “Patient-Centric Decision Intelligence”

**DOI:** 10.7759/cureus.100323

**Published:** 2025-12-29

**Authors:** Girik Subudhi, Shradhit Subudhi, Snigdh Garg, Dishita Mehandru

**Affiliations:** 1 Medical School, Rohilkhand Medical College and Hospital, Bareilly, Bareilly, IND; 2 Independent Research, Masters in Information Technology and Analytics, Rutgers University, Chicago, USA; 3 Medical School, Subharti Medical College and Chhatrapati Shivaji Subharti Hospital, Swami Vivekanand Subharti University, Meerut, IND

**Keywords:** artificial intelligence (ai), clinical data, clinical data management, healthcare digital ethics, multimodel data, personalized precision medicine

## Abstract

The integration of artificial intelligence (AI) in healthcare is rapidly progressing from diagnostic support to predictive analytics. However, most existing clinical AI systems remain limited to generating predictions without translating them into individualised, actionable decisions for patients. This abstract highlights the need for transitioning from predictive AI models to patient-centric decision intelligence, which contextualizes multimodal patient data to support personalized clinical decision-making. Conventional AI models rely primarily on structured data and image-based inputs. In reality, patient care is influenced by diverse variables, including free-text clinical notes, voice biomarkers, wearable sensor output, social determinants of health, environmental exposures, and physician reasoning. Multimodal deep learning platforms integrating these heterogeneous data streams can deliver context-aware recommendations, reduce diagnostic delays, and support dynamic, individualized treatment plans. Moving toward Decision-Intelligence Healthcare Systems (DIHS) would enable explainable risk stratification, adaptive therapeutics, and real-time bedside guidance. Ethical imperatives-algorithmic transparency, dataset diversity, secure interoperability, and shared clinical accountability must guide deployment to ensure equity and physician trust. The next evolution of AI in medicine lies in decision intelligence, where algorithms function as ethical, explainable, and context-sensitive clinical thinking partners. Such systems can advance precision medicine, particularly in low-resource settings, by improving the quality of care, reducing error rates, and supporting equitable access to personalized treatment.

## Editorial

Artificial intelligence (AI) is transitioning from being a diagnostic adjunct to an integral analytical partner within clinical practice. Although significant progress has been made in image interpretation, risk scoring, and predictive modelling, most existing systems merely predict outcomes rather than support individualized, actionable clinical decisions. This limitation reinforces the need for AI to evolve into patient-centric decision intelligence (DI) systems capable of synthesizing complex multimodal data to guide context-appropriate care.

Most currently deployed clinical AI tools rely heavily on structured datasets, including laboratory values, radiological images, and standardized electronic health record (EHR) fields. However, a substantial proportion of clinically meaningful information resides in unstructured or semi-structured formats, such as free-text clinical notes, discharge summaries, voice and speech patterns, patient-reported outcomes, and longitudinal wearable sensor data [1,2]. Moreover, social determinants of health, such as socioeconomic status, education, housing stability, and environmental exposures, significantly influence disease risk, treatment adherence, and outcomes, yet remain underutilized in conventional AI pipelines [3]. Multimodal deep learning architectures, capable of integrating heterogeneous data streams, offer a pathway to overcome these limitations by enabling holistic patient representations and context-aware recommendations [4].

DI systems extend beyond prediction by embedding clinical reasoning frameworks, causal inference, and dynamic feedback loops into AI models. Such systems can provide explainable risk stratification, suggest adaptive treatment pathways, and update recommendations in real time as patient conditions evolve [5]. For example, rather than merely predicting the likelihood of disease progression, a DI system could recommend personalized diagnostic strategies, prioritize interventions based on patient preferences and resource availability, and highlight trade-offs to support shared decision-making between clinicians and patients [6]. This paradigm is particularly relevant in high-burden and resource-limited settings, where clinician shortages and high patient volumes increase the risk of diagnostic delays and medical errors [7].

Despite their promise, the deployment of Decision-Intelligence Healthcare Systems (DIHS) raises critical ethical, legal, and social challenges. Algorithmic bias arising from non-representative training datasets may exacerbate existing health inequities if not proactively addressed [8]. Transparency and interpretability are essential to foster clinician trust and enable meaningful oversight, especially in high-stakes clinical decisions [9]. Furthermore, questions surrounding data governance, patient privacy, interoperability, and shared medico-legal accountability between clinicians and AI systems remain unresolved [10]. Ethical frameworks emphasizing fairness, explainability, accountability, and human-in-the-loop governance must therefore guide the development and implementation of DI-based models.

In this context, AI should be conceptualized not as a replacement for clinicians but as a cognitive augmentation tool that enhances human judgment. By offloading data-intensive synthesis tasks and providing evidence-informed recommendations, DI can allow clinicians to focus on empathy, communication, and complex ethical reasoning elements of care that remain uniquely human [11]. As medicine enters an increasingly data-driven era, regulated, explainable, and equitable DIHS have the potential to accelerate precision medicine, reduce preventable errors, and improve the quality and consistency of care across diverse healthcare systems.
